# Supplementary material for: The Therapeutic Potential of West Indian Lemongrass (Cymbopogon citratus) Essential Oil-Based Ointment in the Treatment of Pitted Keratolysis
Source: Antibiotics (Basel). 2025 Feb 27;14(3):241. doi: 10.3390/antibiotics14030241 (PMC11939757; doi:10.3390/antibiotics14030241)
Supplement: Supplementary file 1 [file antibiotics-14-00241-s001.zip › antibiotics-3416675-supplementary.pdf]

Supplementary data S1.

Statistical analysis - ANOVA

There is a significant difference between the creams, the bacteria and the applied concentrations, so we performed the Post-hoc analysis with Tukey's range test to see exactly where the difference occurs. JASP0.18.3.0.

Three parallel measurements were carried out in order to test the antibacterial efficacies of the different ointment bases, containing 1%, 3% and 5% LEO concentrations. For the statistical analysis, resulst of these 3 experiments were used. A value of $p < 0.05$ was considered significant. During the analysis, the CFU was the dependent variable, while the fixed factors were bacteria, the concentration and the types of the ointments. P-value was <0.01 by the following criteria: type of ointments, bacteria and concentrations, moreover by the type of oitments*bacteria, type of ointments*contentration, bacteria*concentration, and type of ointments*bacteria*contentration. The post-hoc analysis was a comparison of bacteria*ointment type*concentration. We used Tukey's test to assess the significance.

Based on that procedure the following establishments could be performed:

In the case of B. thuringiensis PK2021, there is no significant difference between 1% vs 3%, 1% vs 5% and 3% vs 5% hydrogelum methylcellusose, Ungentum oleosum and vaselinum cholesterinatum, however, a significant difference can be observed in the case of unguentum stearini with 1% essential oil content compared to the ointment bases with 3 and 5% essential oil content. There is no significant difference in the case of unguentum stearini with 3% and 5% essential oil content.

In the case of B. thuringiensis_ E2000/1, a significant difference can be observed between 1% and 3% hydrogel methylcellulose, and a significant difference can also be observed between 3% and 5% hydrogel methylcellulose. There is no significant difference between the 1% essential oil-containing Ungentum oleosum ointment base and 3% and 5%, and there is no significant difference between 3% and 5%. In the case of Ungentum stearini and Vaselinum cholesterinatum ointment bases, there is no significant difference between 1% vs 3%, 1% vs 5% and 3% vs 5%.

For B. thuringiensis_ E2000/2, there is no significant difference between 1% vs 3%, 1% vs 5% and 3% vs 5% for hydrogel methylcellulose, Ungentum oleosum and Vaselinum cholesterinatum ointment bases. For Ungentum stearini ointment base, there is a significant difference between 1% vs 3 and 1% vs 5%, but there is no significant difference between 3% vs 5%.

For B. thuringiensis_ E2008/6, there is a significant difference between 1% vs 3%, 1% vs 5% for hydrogel methylcellulose ointment base, while there is no significant difference between 3% vs 5%. In the case of Ungentum oleosum, there is no significant difference between 1 vs 3, 1 vs 5% and 3 vs 5%. In the case of Ungentum stearini, there is no significant difference between 1 vs 3%, while there is between 1 vs 5% and 3% vs 5%. In the case of Vaselinum cholesterinatum, there is no significant difference between 1 vs 3%, 1 vs 5% and 3 vs 5%.

B. thuringiensis_ E2008/7 There is a significant difference between 1% vs 3%, 1% vs 5% hydrogelum methylcellusose and, while there is no difference between 3% vs 5%. In the case of Ungentum oleosum, there is no significant difference between 1 vs 3 and 1 vs 5%, 3% vs 5%. There is no significant difference between Ungentum stearini 1 vs 3%, while there is between 1 vs 5% and 3 vs 5%. There is no significant difference between 1vs3%, 1vs5% and 3vs5% of Vaselinum cholesterinatum ointment base

In B. thuringiensis_ E2011/1, a significant difference can be observed in the case of 1vs3, 3vs5% and 3vs5% essential oil hydrogel methylcellulose ointment base, on the other hand, there was no significant difference in ungentum oleosum, ungentum strearini and vaselinum cholesterinatum 1vs3%, 1vs5% and 3vs5%.

In B. thuringiensis_ E2014/1, a significant difference can be observed in the application of 1vs3% and 1vs5% essential oil hydrogel methylcellulose, while there is no significant difference between 3% vs 5%. In the case of Ungentum oleosum and Vaselinum cholesterinatum, there is no significant difference between 1vs 3%, 1vs5% and 3vs5%. There is a significant difference between Ungentum stearini 1vs3% , 1vs5%, while there is no significant difference between 3vs5%.

In the case of B. thuringiensis_ E2016/3, there is a significant difference between hydrogel methylcellulose 1vs3% and 3vs5%, while there is no difference between 1vs5%. In the case of Ungentum oleosum, ungentum stearini and Vaselinum cholesterinatum, there is no significant difference between 1vs3, 1vs5% and 3vs5%.

In the case of B. thuringiensis_ E2017/4, there is a significant difference between hydrogel methylcellulose 1vs3% , 1vs5%, while there is no difference between 3vs5%. In the case of Ungentum oleusum, there is no significant difference between 1vs3%, 1vs5% and 3vs5%. In the case of Ungentum stearini, there is a significant difference between 1vs3% and 3vs5%, while there is no significant difference between 1vs5%. In the case of Vaselinum cholesterinatum, there is no significant difference between 1vs3, 1vs5 and 3vs5%.

In the case of B. thuringiensis_ E2020/2, there is a significant difference between hydrogel methylcellulose and ungentum stearini 1vs3% , 1vs5%, while there is no significant difference between 3vs5%. In the case of Ungentum oleusum and vaselinum cholesterinatum, there is no significant difference between 1vs3%, 1vs5% and 3vs5%.

In the case of B. thuringiensis_ E2020/8, there is a significant difference between hydrogel methylcellulose 1vs3% , 1vs5%, while there is no significant difference between 3vs5%. In the case of Ungentum oleosum, there is no significant difference between 1vs3%, 1vs5% and 3vs5%. In the case of Ungentum stearini, there is no significant difference between 1vs3%, while there is between 1vs5% and 3vs5%. In the case of Vaselinum cholesterinatum, there is no significant difference between 1vs3%, 1vs5% and 3vs5%.

In the case of Dermatophilus congolensis_DSM 44180, there is no significant difference between 1vs3% for hydrogel methylcellulose, while there is between 1vs5% and 3%vs5%. In the case of Ungentum oleosum, Ungentum stearini and Vaselinum cholesterinatum, there is no significant difference between 1vs3%, 1vs5% and 3vs5%.

In the case of Kytococcus sedentarius_DSM 20547, there is no significant difference between 1vs3% for hydrogel methylcellulose, while there is between 1v
